# Prognostic Risk Model of Megakaryocyte–Erythroid Progenitor (MEP) Signature Based on AHSP and MYB in Acute Myeloid Leukemia

**DOI:** 10.3390/biomedicines13081845

**Published:** 2025-07-29

**Authors:** Ting Bin, Ying Wang, Jing Tang, Xiao-Jun Xu, Chao Lin, Bo Lu

**Affiliations:** 1 Department of Haematology, The Seventh Affiliated Hospital, Sun Yat-sen University, Shenzhen 518000, China; bint@mail.sysu.edu.cn (T.B.); wangy935@mail.sysu.edu.cn (Y.W.); tangj26@mail2.sysu.edu.cn (J.T.); 2 Pediatric Hematology Laboratory, Division of Hematology/Oncology, Department of Pediatrics, The Seventh Affiliated Hospital, Sun Yat-sen University, Shenzhen 518000, China

**Keywords:** biomarkers, prognosis, megakaryocyte–erythroid progenitor, acute myeloid leukemia, overall survival

## Abstract

**Background**: Acute myeloid leukemia (AML) is a common and aggressive adults hematological malignancies. This study explored megakaryocyte–erythroid progenitors (MEPs) signature genes and constructed a prognostic model. **Methods**: Uniform manifold approximation and projection (UMAP) identified distinct cell types, with differential analysis between AML-MEP and normal MEP groups. Univariate and the least absolute shrinkage and selection operator (LASSO) Cox regression selected biomarkers to build a risk model and nomogram for 1-, 3-, and 5-year survival prediction. **Results**: Ten differentially expressed genes (DEGs) related to overall survival (OS), six (AHSP, MYB, VCL, PIM1, CDK6, as well as SNHG3) were retained post-LASSO. The model exhibited excellent efficiency (the area under the curve values: 0.788, 0.77, and 0.847). Pseudotime analysis of UMAP-defined subpopulations revealed that MYB and CDK6 exert stage-specific regulatory effects during MEP differentiation, with MYB involved in early commitment and CDK6 in terminal maturation. Finally, although VCL, PIM1, CDK6, and SNHG3 showed significant associations with AML survival and prognosis, they failed to exhibit pathological differential expression in quantitative real-time polymerase chain reaction (qRT-PCR) experimental validations. In contrast, the downregulation of AHSP and upregulation of MYB in AML samples were consistently validated by both qRT-PCR and Western blotting, showing the consistency between the transcriptional level changes and protein expression of these two genes (*p* < 0.05). **Conclusions**: In summary, the integration of single-cell/transcriptome analysis with targeted expression validation using clinical samples reveals that the combined AHSP-MYB signature effectively identifies high-risk MEP-AML patients, who may benefit from early intensive therapy or targeted interventions.

## 1. Introduction

There is no doubt that leukemia has long been the second leading cause of cancer-associated fatality in younger individuals [1]. Acute myeloid leukemia (AML) is the most common acute leukemia in adults and has an incidence of three to five cases per 100,000 people annually [2,3]. As an acute myeloid cell malignancy, immature myeloid cell proliferation is a feature of AML, impairing normal hemopoiesis and resulting in severe infections, anemia, and hemorrhage. A number of patients present with extramedullary disease, such as involvement of the central nervous system [3]. Over the past decades, hematopoietic stem cell transplantation, chemotherapy, and supportive care have improved survival rates for AML significantly. Immunotherapy for AML has explored a number of immune checkpoint molecules as well as their associated targets [4]. Despite the fact that 60% to 85% of young patients achieve complete responses with standard induction regimens, overall long-term survival is restricted by refractory disease and relapse [3,5]. Thus, novel biomarkers that can be employed to predict long-term survival are urgently needed and should be explored.

The megakaryocytic and erythroid lineages originate from bipotent megakaryocyte–erythroid progenitors (MEPs), which differentiate into megakaryocytic (GPA^−^CD41^+^) and primitive erythroid (GPA^+^CD41^−^) lineages [6]. Studies have demonstrated that in AML, expression of the fusion protein CBFβ-SMMHC (CM)—a known disruptor of myeloid and lymphoid differentiation—leads to dysregulation of erythroid and megakaryocytic fate-determining factors. Additionally, it disrupts adult erythropoiesis and generates stress-resistant preleukemic pre-MEPs that are prone to malignant transformation, indicating that MEP abnormalities may serve as an early predictive risk factor for AML [7]. Furthermore, Prdm16s expression in MEPs caused AML by converting MEPs into granulomonocytic cells, which then develop into myeloid leukemia stem cells [8]. Zhang et al. demonstrated that in patients transplanted with NrasG12D/+p53^−/−^ bone marrow cells, p53^−/−^ synergizes with enhanced oncogenic Nras signaling to convert MEPs and promote AML development [9]. However, there is currently no MEP-related risk model to predict the prognosis of AML patients. In contrast, Tina Nilsson et al. created CRISPR/Cas9-engineered induced pluripotent stem cell (iPSC) lines harboring the t(7;12) translocation. Their leukemia model of t(7;12) AML demonstrated inhibited differentiation, resulting in the accumulation of erythroid and myeloid progenitor stages—aligning with findings in MNX1-transduced murine hematopoietic stem and progenitor cells (HSPCs) that displayed significant differentiation arrest and accumulation at the megakaryocyte–erythroid progenitor (MEP) stage [10]. Such findings indicate that downregulation or insufficient activation of relevant genes are typical in AML, highlighting the need for drugs to overcome this differentiation block. These research gaps offer a trajectory for our study.

Advancements in single-cell RNA sequencing (scRNA-seq) have enabled the characterization of transcriptome patterns at the cellular level, demonstrating significant efficacy for bulk sample evaluation. Peter van Galen et al. analyzed stemness, developmental hierarchy, and the interactions between malignant cells and immune cells in AML with scRNA-seq technology, revealing the links between AML grade, disease progression, and immunity [11]. Multiple studies indicate that MEPs play an essential role in AML. Therefore, this study aimed to identify markers that are connected with MEPs and to assess their prognostic significance in AML.

## 2. Materials and Methods

### 2.1. Source of Data

In this article, keywords such as “acute myeloid leukemia” and “bone marrow aspirates” were entered into the GEO search box to collect publicly available datasets analyzed by the experiment. The GEO database (accessible at https://www.ncbi.nlm.nih.gov/geo/, accessed on 28 April 2025) was employed to obtain raw data for bone marrow aspirate samples with single-cell transcriptome profiling from GSE116256 (16 AML patients and 6 healthy samples) [11]. The TCGA database (https://www.cancer.gov/ccg/research/genome-sequencing/tcga, accessed on 28 April 2025) was used to gather AML transcriptomes and clinical information. There were 132 patients for whom clinical and survival data were available (Dead: 80, Alive: 52). Patients without follow-up information were omitted from the assessment of survival. GSE71014 data [12] was obtained from the GEO database (https://www.ncbi.nlm.nih.gov/geo/, accessed on 28 April 2025). This study utilized GSE71014 data derived from the GPL10558 HumanHT-12 V4.0 expression bead chip from Illumina, which contained 104 AML patients with full OS information (Dead: 36, Alive: 68) and gene expression data. The TCGA-AML and GSE71014 datasets were employed, respectively, as sets for training and verification. The workflow of this research is shown in Supplementary Figure S1.

#### Information Processing for ScRNA-Seq

The scRNA-seq information [13] was analyzed via the Seurat R package (v4.1.1). We calculated the percentages of gene counts, cell counts, and mitochondria sequence counts using a standard information preprocessing procedure. We excluded cells with fewer than 200 detectable gene counts and genes identified in fewer than 3 cells. The proportion of mitochondria was restricted to 5% or lower. Seurat employed the top 2000 variable genes in the RunPCA function. Uniform manifold approximation and projection (UMAP) investigation was employed to reduce dimensionality and locate clusters. The main cell clusters in UMAP were displayed using a resolution of 0.5. To identify different types of cells in a cluster of data, the SingleR package (v2.10.0) was used [14].

### 2.2. Cell-to-Cell Communication

Seven cell cohorts, including MEPs, CMPs, B cells, NK cells, T cells, monocytes, and GMPs, were examined using the R package “CellChat” (v1.0.0) in order to study the communication interactions between cells and determine the mechanisms of the communicating molecules [15]. The “aggregateNet” function was used to visualize the signals from each cell cohort and compute aggregated cell–cell communication networks. The “identifyCommunicationPatterns” function allows for the identification of incoming and outgoing signals from specific types of cells as well as the visualization of signaling pathways. The “computeNetSimilarity” function was used to confirm signaling cohorts based on how similar their functional and structural characteristics were.

### 2.3. Differential Expression Assessment Using ScRNA-Seq

To compare the differential gene expression for healthy control and AML cohorts of the MEPs, the “FindMarkers” function in Seurat was used with default parameters. We used criteria of adj.p less than 0.05 and |Log2fold change| more than 1. The R packages “pheatmap” and “ggplot2” were used to create a heatmap and a volcano plot, respectively [16].

### 2.4. GO and KEGG Analyses

Gene Ontology (GO) falls under the following three headings: biological process (BP), cellular component (CC), and molecular function (MF). With the help of the R package “clusterProfiler” (v4.4.4), the Kyoto Encyclopedia of Genes and Genomes (KEGG) pathway evaluation and the GO evaluation of the differentially expressed genes (DEGs) [17] were completed.

### 2.5. Formation and Validation of a Prognostic Model Based on DEGs

Genes significantly associated with overall survival in the TCGA-AML training cohort were identified by univariate Cox regression analysis. Aiming to address potential multicollinearity and overfitting issues inherent in high-dimensional genomic data, the least absolute shrinkage and selection operator (LASSO) regression analysis was used on the candidate genes identified via univariate Cox regression. The optimal regularization parameter was determined through 10-fold cross-validation and selected based on the minimum criterion, corresponding to the point where the cross-validated mean squared error (MSE) reached its lowest value. This approach ensures a balance between model complexity and predictive accuracy, effectively shrinking the coefficients of less relevant variables to zero. The prognostic risk score’s calculation was as follows:

(1) $$Risk\;score\;equals\;to\;\left(\beta A\;\times \;Gene\;A\;expression\right)\;plus\;\left(\beta B\;\;\times \;Gene\;B\;expression\right)\ldots \;\;\;\;+\left(\beta N\;\times \;Gene\;N\;expression\right)$$

β is its coefficient. To categorize TCGA-AML patients into both high- and low-risk cohorts, the median risk score was used. The log-rank test and a Kaplan–Meier (K-M) survival curve were employed to evaluate the differences in cohort survival rates. ROC curve analysis was employed to assess the prognostic performance and validate the previously indicated findings. The validation cohort comprised GSE71014. The Wilcoxon rank-sum test was employed to compare various clinicopathological variables.

### 2.6. Developing and Validating a Nomogram

In this study, independent prognostic indicators for AML were examined using univariate and multivariate Cox regression analyses on the TCGA dataset. A nomogram was developed based on the outcomes of univariate and multivariate Cox regression analyses to predict the 1-, 3-, and 5-year survival rates of AML patients. The anticipated survival outcome was subsequently compared to the actual outcome using calibration curves, and the clinical utility of the nomogram was assessed through decision curve analysis (DCA).

### 2.7. Single-Cell Trajectory Analysis

To deduce the gene regulatory events that drive the transition between cellular states, we used the Monocle R package (v2.36.0) [18] to construct a single-cell pseudotime trajectory of scRNA-seq data. The plot cell trajectory function facilitates the visualization of cell trajectory inference by employing pseudotime and cell type.

### 2.8. Using Quantitative Real-Time Polymerase Chain Reaction (qRT-PCR) for Validation

In this study, a total of 10 paired samples of patients from the Seventh Affiliated Hospital, Sun Yat-Sen University were utilized for qRT-PCR; the collecting process lasted from 5 March 2024 to 5 March 2025. A detailed summary of patient demographic data is exhibited in Supplementary Table S1. The study was approved by the Ethics Committee of Seventh Affiliated Hospital, Sun Yat-Sen University (KY-2024-299-01). Written informed consent to participate in this study was provided by the participants. All procedures involving human participants were performed in accordance with the Declaration of Helsinki. QRT-PCR was used to confirm the expression of the signature genes involved. Total RNA was extracted from 10 bone marrow aspirates with AML and 10 control samples using TRIZol (Solarbio, Beijing, China). Using a NanoDrop 2000 spectrophotometer (Thermofisher, Durham, NC, USA), the obtained RNA samples’ quantity and purity were assessed. In addition to reverse transcription of mRNA into cDNA, qPCR reactions were carried out using 2 µL cDNA, 10 µL of SYBR PCR mix (Yeasen, Shanghai, China), 6.8 µL ddH_2_O, and 0.4 µL ROX Reference Dye2. Using Primer 5.0, primer sequences for signature genes were designed and are indicated in Table 1. Signature genes’ relative expression levels were calculated under the specified thermal cycling conditions using the 2^−△△Ct^ method.

### 2.9. Western Blotting for the Verification of Post-Transcriptional Expression

Utilizing high sensitivity and antibody specificity, Western blotting was conducted to confirm the protein expression levels of signature genes, therefore mitigating interference from post-transcriptional expression. A total of 300 μL of whole blood was combined with 300 μL of protein extract, agitated at 4 °C for 30 min, and centrifuged at 14,000× *g* at 4 °C for 10 min, and the supernatant was transferred to a clean EP tube to isolate whole blood proteins. The protein concentration was quantified with the BCA Protein Assay Kit (P0010, Beyotime, Shanghai, China). The protein solution was mixed with 5× protein loading buffer (Servicebio, G2075-100ML, Wuhan, China) at a ratio of 4:1 thoroughly, followed by heating the resultant mixture in a 95 °C metal bath for 10 min to ensure complete protein denaturation. After cooling to room temperature, the sample was stored at −20 °C or −80 °C for subsequent use. For SDS-PAGE electrophoresis, separating and stacking gels were prepared according to the molecular weight of the target proteins. The stacking gel was run at a constant voltage of 80 V for approximately 30–40 min; the separating gel was run at a constant voltage of 120 V until the pre-stained protein marker (bromophenol blue) migrated to the bottom of the gel. Subsequently, the gel and PVDF membrane were immersed in a cold bath while maintaining a continuous current of 200 mA for one hour. During the immunoreaction, the membrane was washed with TBST and incubated with 5% skimmed milk powder for 30 min. The primary antibody (MYB Rabbit pAb, A13776-50 μL; AHSP Rabbit pAb, A6465-50 μL; Abclonal, Wuhan, China) was diluted according to the instructions and incubated overnight at 4 °C, whilst the secondary antibody (Goat anti-Rabbit IgG (H + L) Secondary Antibody, HRP, 31460, Invitrogen), diluted at 1:5000, was applied and incubated at room temperature for 30 min. Following incubation, the membranes were treated with ECL chemiluminescent substrate and visualized using a chemiluminescence imaging system (GelView 6000 Plus, bltlux, Guangzhou, China). Image analysis was performed utilizing Image J software (bundled with 64-bit Java 8.), where the relative protein expression was calculated as the ratio of the target protein’s band intensity to that of the loading control.

## 3. Results

### 3.1. Assessment of ScRNA-Seq Data and Identification of Genes Associated with MEP

**Figure 1:**
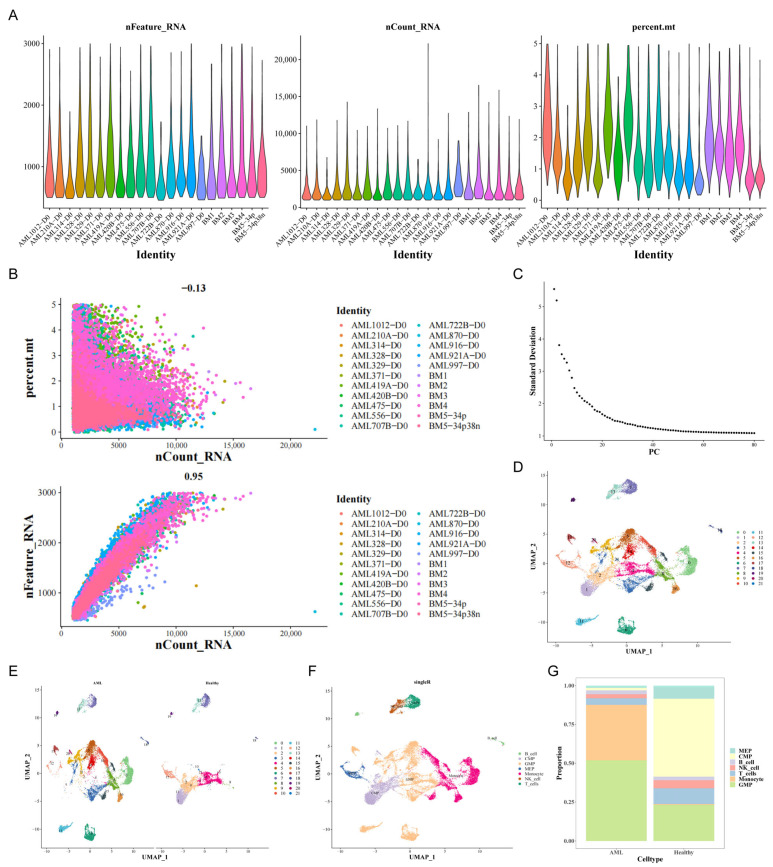
Characteristics of single-cell RNA sequencing (scRNA-seq) from AML samples and identification of 7 cell types. (**A**) Quality control of scRNA-seq from 16 AML samples. (**B**) The detected gene numbers were positively correlated with the sequencing depth. Pearson’s correlation coefficient reached 0.95. (**C**) Principal component analysis (PCA) analysis for dimensionality reduction of the top 2000 variable genes. (**D**) Uniform manifold approximation and projection (UMAP) plot of the analyzed single cells. Each color represents one cluster. (**E**) UMAP visualization between AML and healthy samples. (**F**) UMAP plot of 7 identified main cell types in AML. (**G**) The distribution of cell types between AML and healthy samples.

A quality control chart is indicated in Figure 1A showing the range of detected gene amounts and the sequencing count of each cell. After quality control, 22,395 cells and 19,075 genes were employed for downstream assessment. In addition, as shown in Figure 1B, we observed a significant positive correlation between the detected gene amounts and sequencing depth (equals to 0.95). Initially, by employing principal component analysis (PCA) on the top 2000 variable genes, we successfully reduced the dimensionality of the data (Figure 1C)**,** subsequently confirming 21 unique clusters by UMAP evaluation (Figure 1D,E). After dimensionality reduction, UMAP was employed to illustrate cell types following cluster annotation using the SingleR tool (v2.10.0). This stage revealed the presence of seven distinct cell types: MEPs, CMPs, B cells, NK cells, T cells, monocytes, and GMPs (Figure 1F). A histogram depicting the percentage of different cell types within each cohort of AML and control cells was utilized to elucidate the cellular makeup (Figure 1G).

### 3.2. An Assessment of the Interaction Between Cells in Patients with AML

Based on the consolidated cell–cell contact network illustrated in Supplementary Figure S2A,B, notable alterations in cell types and interaction intensity were detected. We found that MEPs possessed a comprehensive connectivity network with other clusters. MEPs showed the highest levels and strengths of interaction with B cells and monocytes [19]. Supplementary Figure S2C,D categorizes numerous signaling pathways based on their functional and structural similarities. A functional similarity cohort was employed to identify a cohort of pathways. Cohort 1 is predominantly characterized by BAFF and MIF pathways. Relying on the communication patterns, two outgoing signaling patterns were validated (Supplementary Figure S2E) and two incoming signaling patterns were confirmed (Supplementary Figure S2F). For incoming signaling patterns of target cells, pattern 1 showed the crosstalk among B cells, while pattern 2 mainly gathered the communications of GMPs and monocytes. We also found that pattern 1 expressed the BAFF signaling pathways, while pattern 2 expressed the MIF signaling pathways. For outgoing signaling patterns of secreting cells, pattern 1 mainly gathered the communications of B cells, CMPs, and MEPs; pattern 2 mainly gathered the communications of GMPs and monocytes. We also found that pattern 1 expressed the MIF signaling pathways, while pattern 2 expressed the BAFF signaling pathways [20]. We analyzed the cell–cell interactions across particular pathways throughout various stages (Supplementary Figure S3A,B). Supplementary Figure S3C illustrates the MIF signaling pathway network in AML. Supplementary Figure S3D shows the BAFF signaling pathway network in AML.

### 3.3. Identifying DEGs

**Figure 2:**
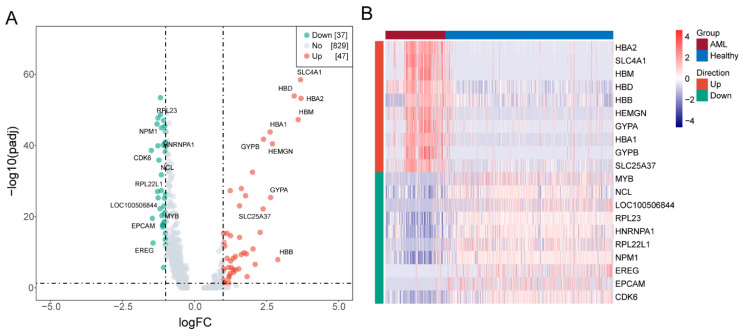
Identification of differentially expressed genes (DEGs) associated with MEPs between the healthy control and AML groups. (**A**) Volcano map of DEGs between the two groups. A red dot represents an upregulated gene, and a green dot represents a downregulated gene. (**B**) Heatmaps of DEGs.

As a result of the screening, 84 DEGs were confirmed (AML-MEP versus healthy control-MEP). In total, 37 genes were downregulated compared to 47 being upregulated (Figure 2A). In Figure 2B, a heatmap depicting the expression of DEGs is displayed.

### 3.4. Analyses of Functional Enrichment

**Figure 3:**
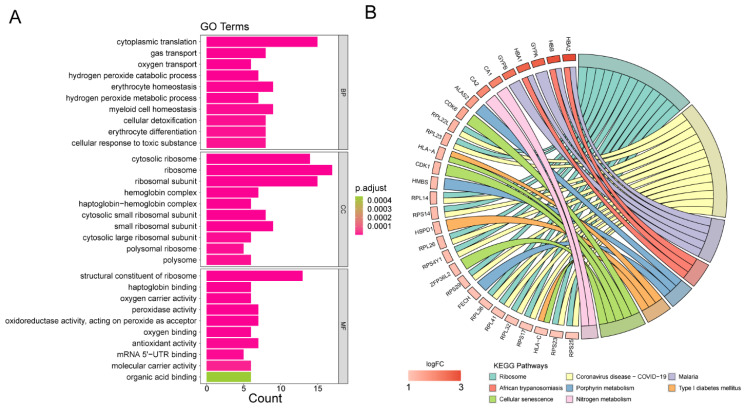
Function enrichment analyses of DEGs involved. (**A**) Bar chart of Gene Ontology (GO) analysis. (**B**) Circle plot of Kyoto Encyclopedia of Genes and Genomes (KEGG) analysis.

A summary of the outcomes of GO and KEGG enrichment assessment is provided in Figure 3A,B. According to the GO assessment of DEGs, they are highly enriched in cytoplasmic translation, transport of gas, cytosolic ribosomes, ribosomes, structural constituents of ribosomes, haptoglobin binding, etc. As a result of the KEGG assessment of DEGs, they were primarily enriched in ribosomes and coronavirus disease—COVID-19.

### 3.5. The Development of a Risk Model

**Figure 4:**
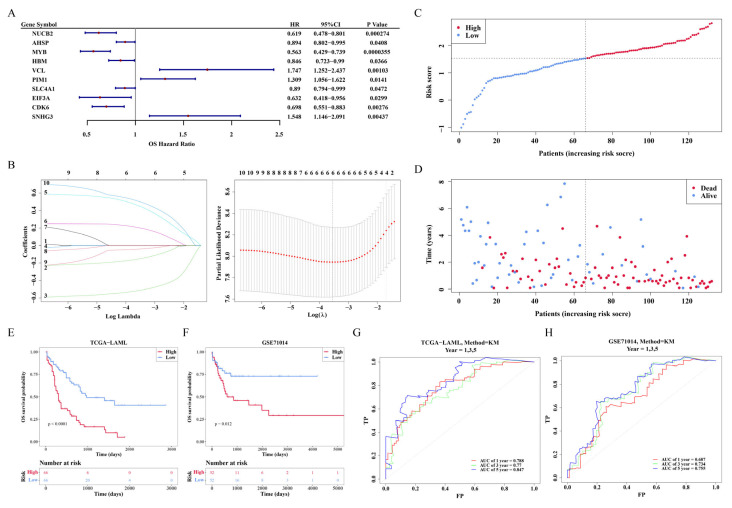
Construction and validation of the risk model. (**A**) Univariate Cox regression analysis for 10 overall survival (OS)-related DEGs. (**B**) The least absolute shrinkage and selection operator (LASSO) analysis to construct a risk model. (**C**) The risk curve based on the risk score of each sample. (**D**) The scatter plot based on the survival status of each sample. The green and red dots represent survival and death, respectively. (**E**) Kaplan–Meier (K-M) survival curves between high- and low-risk groups in the TCGA-AML dataset. (**F**) K-M survival analysis in GSE71014. (**G**) ROC curves for predictive accuracy of the risk model in the TCGA-AML dataset. (**H**) ROC curves analysis in GSE71014.

Relying on the outcomes of the univariate Cox regression assessment (Figure 4A), a total of 10 DEGs have been confirmed as being significantly related to OS. In addition, six genes (AHSP, MYB, VCL, PIM1, CDK6, as well as SNHG3) were investigated using the LASSO regression method (λ = 0.0286) (Figure 4B).

The following equation was used to determine risk scores:

Score for risk equals to minus 0.040 × AHSP expression minus 0.514 × MYB expression plus 0.455 × VCL expression plus 0.204 × PIM1 expression minus 0.153 × CDK6 expression plus 0.505 × SNHG3 expression.

On the basis of the median risk ratings for each AML patient, the samples from the TCGA-AML cohort were split into two cohorts (Figure 4C). The risk plot demonstrated that patients with high risk ratings had shorter survival periods than those with low risk scores (Figure 4D). The K-M curves in the TCGA-AML cohort revealed that patients in the high-risk group had a worse overall survival rate (*p* < 0.0001) (Figure 4E). In the GSE71014 validation cohort, the low-risk cohort’s survival rate was greater (Figure 4F) (*p* equal to 0.012). The areas under the ROC curve (AUCs) for OS in the training group were 0.788, 0.770, and 0.847 for the 1-, 3-, and 5-year intervals, respectively (Figure 4G). The AUCs for OS in the validation cohort were 0.687 at 1 year, 0.734 at 3 years, and 0.755 at 5 years (Figure 4H). These findings involving the risk scores may enhance personalized clinical decision-making by allowing for the early identification of high-risk AML patients who could benefit from more aggressive therapies, while enabling low-risk patients to evade unnecessary overtreatment, thus optimizing treatment efficacy and improving quality of life.

### 3.6. Nomogram Construction

**Figure 5:**
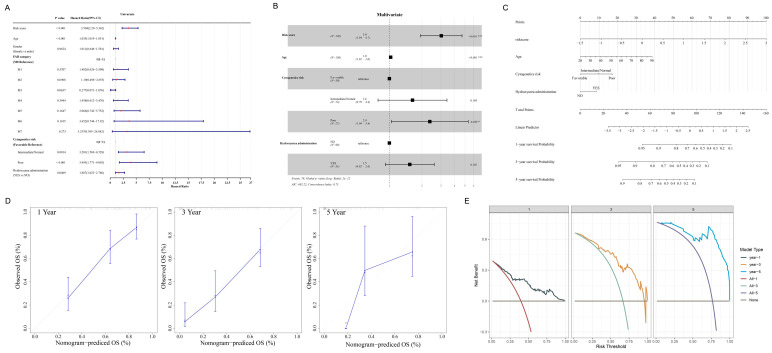
Establishment and evaluation of nomogram. (**A**) Forest plot of univariate Cox regression analysis. (**B**) Forest plot of multivariate Cox regression analysis. (**C**) Nomogram was constructed for survival prediction of AML patients at 1, 3, and 5 years. (**D**,**E**) Construction of calibration curve (**D**) and decision curve analysis (DCA) (**E**) for assessing the predictive efficiency of nomogram model. * *p* < 0.05; *** *p* < 0.001.

Based on the results of a univariate analysis, risk score, age, cytogenetic risk, and hydroxyurea administration were substantially associated with survival in AML patients (*p* less than 0.05) (Figure 5A). Relying on multivariate assessment (Figure 5B), the risk score and prognosis remained significantly associated (*p* less than 0.05). To predict the survival outcome for AML for one, three, and five years, a nomogram incorporating the risk score, age, cytogenetic risk, as well as hydroxyurea administration was developed (Figure 5C). No deviation was observed between the nomogram-based survival probabilities and actual survival probabilities (Figure 5D). Similarly, the DCA curve in Figure 5E indicated that the nomogram may provide clinical benefits to patients with AML for 1, 3, and 5 years. This nomogram integrates essential prognostic factors into an accessible tool, aiding clinicians in quantifying the survival probabilities of individual AML patients over defined timeframes, thus facilitating more accurate treatment planning and prognostic communication, ultimately improving the consistency and efficacy of clinical management.

### 3.7. The Connection Involving the Risk Score as Well as Clinicopathological Attributes

The Wilcoxon rank-sum test indicated a significant difference in risk scores across the cohorts categorized by age (≤60, >60), FAB category (M0–M7), cytogenetic risk (Favorable, Intermediate/Normal, as well as Poor) (Supplementary Figure S4A–C, *p* less than 0.05). Despite this, the risk score did not differ significantly between genders (males and females) or whether hydroxyurea was administered (YES or NO) (Supplementary Figure S4D,E).

### 3.8. Pseudotime Analysis

**Figure 6:**
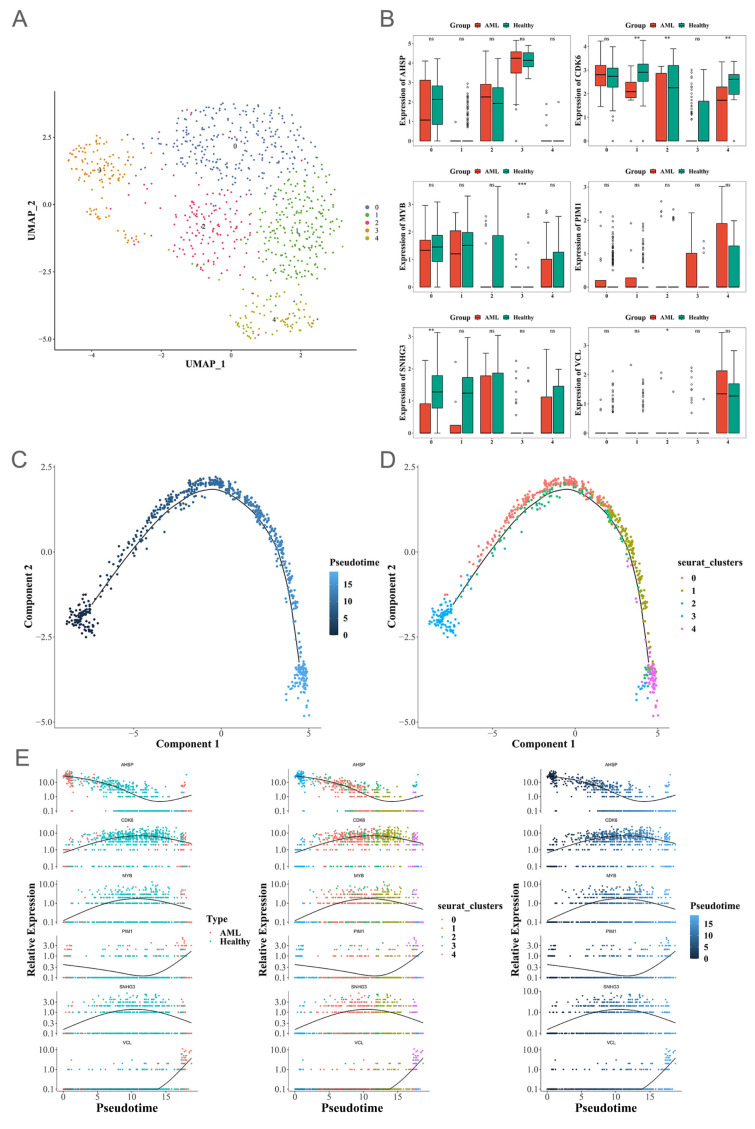
Establishment of pseudotime analysis. (**A**) UMAP clustering of MEP cells. (**B**) Box plots of gene expression levels based on AML and healthy samples for the 5 MEP subtypes. (**C**) MEP cell differentiation time differences. (**D**) Differentiation trajectories of the 5 MEP cell subtypes. (**E**) Time-series gene expression profiles of MEP cells. ns: *p* > 0.05; * *p* < 0.05; ** *p* < 0.01; *** *p* < 0.001.

UMAP clustering was employed to disentangle the heterogeneous landscape of MEP cells, which enabled us to stratify these progenitors into five distinct subclusters (Figure 6A). From Figure 6B, we can see that the CDK6 gene is significantly downregulated in stages 1, 2, and 4. The downward trend of MYB is more prominent in stage 3; the significant change of the SNHG3 gene is detected in stage 0. Pseudotime analysis was integrated to trace the dynamic expression of biomarkers during MEP differentiation (Figure 6C,E), and revealed that the third stage initiates responses at the early stage of differentiation, accompanied by downregulation of MYB; in contrast, the fourth stage mainly activates responses at the terminal stage, characterized by downregulation of CDK6 expression.

### 3.9. Two Signature Genes That Are Validated Using Expressions

**Figure 7:**
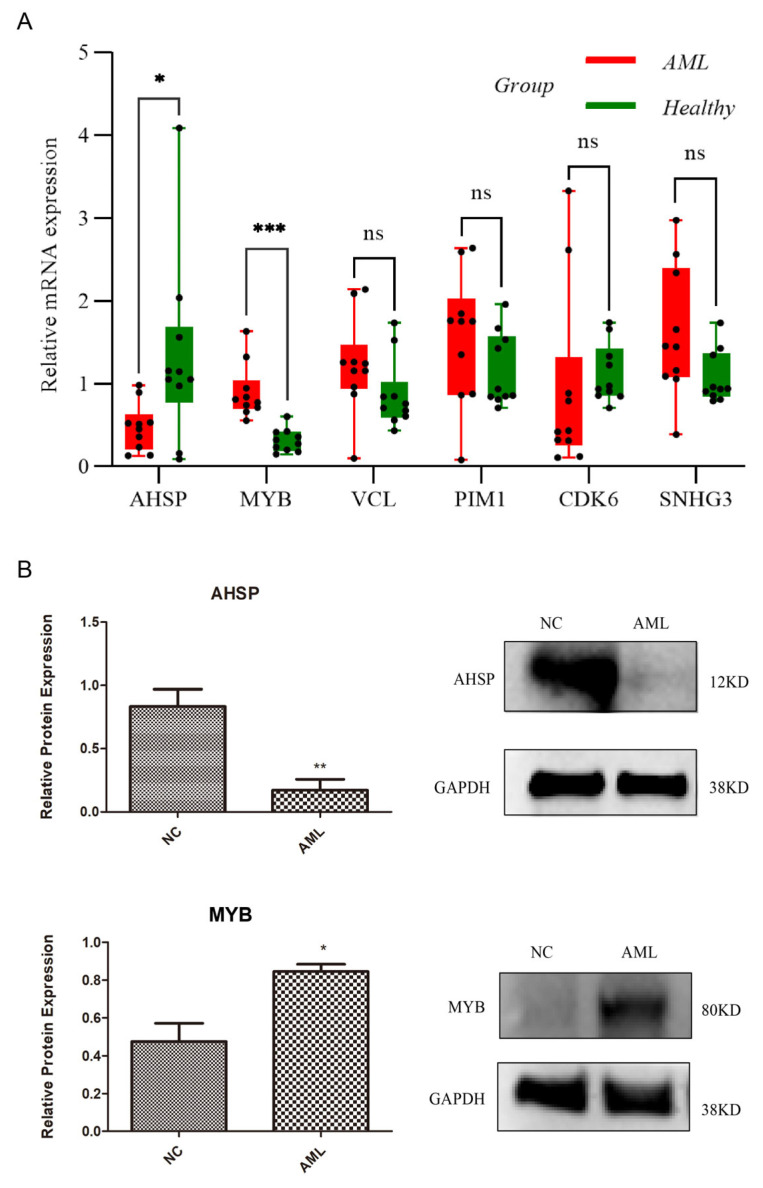
The differential expression levels of AHSP and MYB were validated between the healthy group and AML group of the MEP. (**A**) Quantitative real-time polymerase chain reaction (qRT-PCR). (**B**) Western blotting. ns: *p* > 0.05; * *p* < 0.05; ** *p* < 0.01; *** *p* < 0.001.

Figure 7A illustrates the expression levels of six signature genes using qRT-PCR. AML patients exhibited significant AHSP downregulation and MYB upregulation compared to healthy controls (*p* < 0.05). However, the remaining four signature genes showed no significant differences between the two groups (*p* > 0.05). Subsequently, Western blotting confirmed that these two genes exhibited the same expression changes at the protein level (*p* < 0.05), verifying the consistency between the transcriptional level changes and protein expression of AHSP and MYB (Figure 7B).

## 4. Discussion

AML represents a clonal tumor originating from hematopoietic stem cells, characterized by an aggressive clinical trajectory, poor prognosis, and uncontrolled proliferation of immature myeloid cells [3,21]. Pathogenetic mechanisms involve dysregulated differentiation of MEPs, which shows strong correlations with erythroid/megakaryoblastic AML variants. These MEP-associated AML subtypes exhibit resistance to conventional chemotherapy and dismal survival rates, primarily attributed to BCL-XL-mediated survival mechanisms and recurrent TP53/IDH1 genetic alterations, highlighting the urgent need for BCL-XL-targeted agents and combined immunotherapeutic regimens to enhance clinical outcomes [22,23]. While these pathobiological features carry significant therapeutic implications, existing prognostic frameworks remain deficient in integrating MEP-associated molecular indicators.

In this study, our model—constructed by integrating single-cell transcriptome data with the TCGA-LAML cohort—exhibits moderately high predictive accuracy (AUC > 0.68) and clinically acceptable discriminative ability. Patient stratification by risk scores revealed significant survival differences, with validation in the GSE71014 dataset confirming its potential clinical utility for AML forecast. A key advantage is its ability to capture MEP-related biological features often overlooked by conventional systems, offering a novel tool for precise AML subtyping. Notably, integration with traditional ELN/LSC17 scores might enhance identification of “cryptic high-risk” patients (e.g., those with favorable cytogenetics but high-risk MEP markers)—a subgroup notoriously challenging to stratify using existing prognostic tools alone. UMAP clustering and pseudotime analysis were integrated to trace the dynamic expression of biomarkers during MEP differentiation. This supplements the machine learning-screened biomarkers with both subtype specificity and differentiation regulatory significance, thereby enhancing their reliability as prognostic indicators. Such computational modeling techniques have become increasingly prevalent in contemporary hematological studies [24,25]. Systematic evaluation (qRT-PCR and Western blotting) further identified substantial downregulation of AHSP (α-hemoglobin stabilizing protein), accompanied by increased MYB (Myeloblastosis oncogene) expression in AML specimens. It echoes the critical associations of these two biomarkers with leukemogenesis: the MYB proto-oncogene contributes to the survival of leukemia stem cells, while AHSP is essential for erythroid differentiation.

Functioning as an erythroid-specific molecular chaperone, AHSP executes critical biological functions through stabilization of unbound α-globin chains to inhibit oxidative damage, promotion of hemoglobin assembly, and regulation of erythroblast maturation processes. In diagnostic pathology, AHSP has been established as a specific immunohistochemical marker in bone marrow evaluation for differentiating acute erythroid leukemia (AEL) from alternative AML subtypes [26,27]. Diminished AHSP expression in AML disrupts normal erythroid development and accelerates malignant transformation of MEPs [28]. Recent investigations by Zhu et al. [29] delineate a pathogenic cascade wherein FLT3-ITD mutations promote leukemogenesis via STAT5/MAPK pathway activation, with AHSP levels demonstrating an inverse relationship with FLT3 expression. Compromised AHSP activity may amplify FLT3-ITD-driven oncogenic signaling, fostering cellular proliferation and apoptotic resistance. These discoveries underscore AHSP’s potential as a diagnostic biomarker and therapeutic target for AML subtype classification and personalized intervention approaches.

MYB functions as a central transcriptional coordinator in AML pathogenesis, governing leukemogenesis initiation and disease persistence by enhancing LICs’ self-renewal capacity and viability through stimulation of downstream tumorigenic pathways involving MYC, CDK6, BCL2, and MCL1 [30]. Molecularly, MYB directly interacts with SE domains to maintain elevated expression of targets like ERG and MYC, while cooperating with chromatin-modifying factors EP300/CBP to amplify transcription [31,32]. Subtype-selective reliance on MYB becomes evident: MLL-rearranged and t(8;21) leukemias depend strongly on MYB (with its ablation inducing differentiation and growth arrest), whereas complex karyotype AML frequently develops compensatory mechanisms. MYB-driven oncogenesis is further reinforced by repressing MAFB, whose re-expression counteracts malignant characteristics in MLL-rearranged models [33]. Additionally, MYB synergizes with C/EBPβ to regulate BCL2 and CDK6-mediated survival pathways, with C/EBPβ inhibition showing substantial therapeutic efficacy [30]. Clinically, MYB hyperactivation correlates with poor outcomes, particularly in MLL-rearranged AML, where MYB-dependent cases have shorter survival. It also promotes chemoresistance through bimodal mechanisms: activating DNA-PK and inducing anti-apoptotic MCL1 [34,35]. These cumulative observations position MYB as a master regulatory node in AML biology, perpetuating LSC functionality through integrated control of oncogenic cascades while emerging as a stratification marker for treatment-refractory disease and therapeutic targeting opportunities. In our analytical results, low expression of MYB in early differentiation-responsive MEP subtypes (stage 3) aligns with its inhibitory potential in normal erythropoiesis, while its upregulation in both mRNA and protein levels in AML samples indicates a dysregulated regulatory shift. These findings reveal the multi-level regulatory characteristics of MYB in AML pathogenesis and its stage-specific role in leukemogenesis and progression, which warrant further elucidation through dynamic single-cell sequencing analyses.

Following systematic analysis of scRNA-seq datasets from AML specimens, we identified and validated pivotal DEGs by contrasting genomic signatures between leukemic cell populations and normal MEPs. Functional annotation indicated enrichment of these markers in ribosome-associated pathways. Ribosomal dysfunction, as a critical determinant in AML pathogenesis, progression, and treatment resistance, has emerged as a therapeutic target, supported by mechanistic links to leukemogenesis involving aberrant ribosomal biosynthesis, translational regulation, and ribosomal protein (RP) mutations. Zhou et al. [36] revealed that variable 2′-O-methylation patterns in LSCs reconfigure translational mechanisms to favor synthesis of codon-optimized transcripts (e.g., nutrient transporters), sustaining LSC proliferation. KMT2D ablation in leukemic cells induces mTOR-mediated nucleolar hyperactivity, characterized morphologically by nucleolar hypertrophy and elevated rRNA production, sensitizing cells to RNA polymerase I inhibitor CX-5461 [37]. Clinically, individuals with Diamond–Blackfan anemia (DBA) harboring RPS/RPL genomic alterations demonstrate increased predisposition to myeloid malignancies [38], underscoring ribosomopathy-driven leukemogenesis. Second, disordered translational initiation mechanisms promote neoplastic protein overproduction in AML. Investigations by Sun et al. [39] characterized the micropeptide APPLE as a leukemic accelerator that stabilizes eIF4F complexes and facilitates mRNA circularization, enhancing translation initiation. RUNX1 mutant cells display enhanced vulnerability to translation inhibitor HHT secondary to ribosomal maturation defects and c-Myc suppression, with synergistic cytotoxicity when combined with BCL2 inhibitors [40]. Single-cell resolution analyses further delineated associations between upregulated RPS transcript levels in leukemic progenitors with treatment resistance and poor remission rates [41]. Synthesizing the existing literature with our experimental observations, we posit that ribosomal dysregulation constitutes a central mechanistic component in AML evolution, potentially sustaining malignant characteristics through deranged proteostasis, metabolic adaptation, and stem cell maintenance. This conceptual framework provides critical insights for designing ribosomal pathway-directed therapeutic strategies in hematologic malignancy management.

Following an extensive literature evaluation and integrative data interpretation, we postulate that coordinated AHSP suppression and MYB transcriptional upregulation may drive clonal propagation in AML via ribosomal dysregulation mechanisms, thereby exacerbating adverse clinical trajectories. Critical methodological constraints merit acknowledgment: Primarily, although VCL, PIM1, CDK6, and SNHG3 showed significant associations with AML survival and prognosis, they did not exhibit pathological differential expression in qRT-PCR experimental validations; thus, mechanistic interrogation through single-cell level functional perturbation assays (e.g., using siRNA or small-molecule inhibitors) remains imperative to delineate precise pathobiological interactions within this regulatory axis. Secondarily, the prognostic analysis of this study relies on public datasets (TCGA-LAML, GSE71014, etc.). Although the robustness of the model has been improved through multi-cohort validation, the sample heterogeneity of public data (such as the completeness of clinical information and differences in detection platforms) may have a certain impact on the interpretation of the results. Meanwhile, the independent experimental validations (qRT-PCR and Western blotting) only focused on the expression changes of key genes, lacking the prognostic correlation validation with large-sample clinical specimens; as part of our future research agenda, we plan to validate the model in multiple independent cohorts and systematically compare its performance with classic prognostic systems—including ELN 2022 criteria, LSC17 score, and other well-recognized gene expression-based signatures. These analyses will specifically evaluate the incremental prognostic value of our model, particularly its ability to refine risk stratification when integrated with existing tools, further clarifying its clinical utility in AML management. Furthermore, while our computational framework offers valuable pathomechanistic inferences, its translational implementation demands optimization through prospective, multi-institutional trials utilizing multi-dimensional omics platforms to augment predictive fidelity and biological coherence.

Overall, the MEP-related AML prognostic model incorporating AHSP and MYB biomarkers exhibits robust prognostic efficacy. For therapeutic targeting, MYB inhibition (e.g., peptidomimetic inhibitors like Celastrol and MYBMIM) [42,43] might disrupt ribosomal dysregulation and leukemic stem cell survival, though it may negatively impact erythroid differentiation. Combining such inhibition with AHSP restoration strategies—such as epigenetic drugs (DNA methyltransferase inhibitors, histone deacetylase inhibitors) [44] or mRNA therapy [45]—effectively improves erythroid differentiation in MEP-AML. Additionally, the model helps select candidates for experimental therapies (e.g., MYB-targeted trials) and avoids overtreatment in low-risk groups, guiding personalized treatment. Ultimately, this work provides a biologically grounded prognostic tool to enhance patient survival.

## 5. Conclusions

In this study, we constructed an AML prognostic prediction framework using public single-cell transcriptome datasets and the TCGA-LAML cohort. UMAP clustering analysis revealed the potential expression profiles of biomarker genes within the MEP cell subsets and confirmed that these biomarkers possess both subtype specificity and differentiation regulatory significance. The expression changes of AHSP-MYB signature were validated by qRT-PCR and Western blotting. After stratifying patients into different risk categories based on risk scores, we observed significant survival differences, and the risk model was further validated. Notably, the combined AHSP-MYB signature effectively identifies high-risk MEP-AML patients, who may benefit from early intensive therapy or targeted interventions.

## Figures and Tables

**Table 1:** Primers for quantitative real-time polymerase chain reaction (qRT-PCR) in the current study.

Primer	Primer Sequence	
	Forward Primer	Reverse Primer
AHSP	TTACAGGCAGCAGGTGACAGG	AGGTGTCAGGGTAGAGTGGCAG
MYB	TCTGCTCACACCACTGGGAAG	ATCTGCCACAGGCGAGGTC
PIM1	CAGGACAGTGCTTGATACAGGAAC	AGAAGAGAGTATCTATGGGAGGAGTT
CDK6	AGTGGTCGTCACGCTGTGGTA	AGGTCCTGGAAGTATGGGTGA
SNHG3	TGCTCAGGAAGAAGCCAGATG	GTAGTTACAGCAGGACACGGATG
VCL	AAGGCCGGGGAGGTGATT	ATGATGTCATTGCCCTTGCTGG
Actin	TGGCACCCAGCACAATGAA	AGGGTGTAACGCAACTAAGTCATAG

## Data Availability

The TCGA-AML dataset was downloaded from the TCGA database (https://www.cancer.gov/ccg/research/genome-sequencing/tcga) (accessed on 13 July 2022). GSE116256 and GSE71014 were derived from GEO database (https://www.ncbi.nlm.nih.gov/geo/) (accessed on 13 July 2022). Further inquiries can be directed to the corresponding author (C.L.).

## Supplementary Materials

The following supporting information can be downloaded at: https://www.mdpi.com/article/10.3390/biomedicines13081845/s1.

## Abbreviations

AML: acute myeloid leukemia; MEP: megakaryocyte–erythroid progenitor; UMAP: uniform manifold approximation and projection; LASSO: least absolute shrinkage and selection operator; DEGs: differentially expressed genes; AUC: area under the curve; OS: overall survival; scRNA-seq: single-cell RNA sequencing; GO: Gene Ontology; BP: biological process; CC: cellular component; MF: molecular function; KEGG: Kyoto Encyclopedia of Genes and Genomes; qRT-PCR: quantitative real-time polymerase chain reaction; PCA: principal component analysis; AEL: acute erythroid leukemia; DBA: Diamond–Blackfan anemia; iPSC: induced pluripotent stem cell; HSPCs: hematopoietic stem and progenitor cells; DCA: decision curve analysis; AHSP: α-hemoglobin stabilizing protein; MYB: Myeloblastosis oncogene.
