# Supplementary material for: Prognostic Risk Model of Megakaryocyte–Erythroid Progenitor (MEP) Signature Based on AHSP and MYB in Acute Myeloid Leukemia
Source: Biomedicines. 2025 Jul 29;13(8):1845. doi: 10.3390/biomedicines13081845 (PMC12383354; doi:10.3390/biomedicines13081845)
Supplement: Supplementary file 1 [file biomedicines-13-01845-s001.zip › biomedicines-3644982-supplementary.pdf]

Supplementary Materials

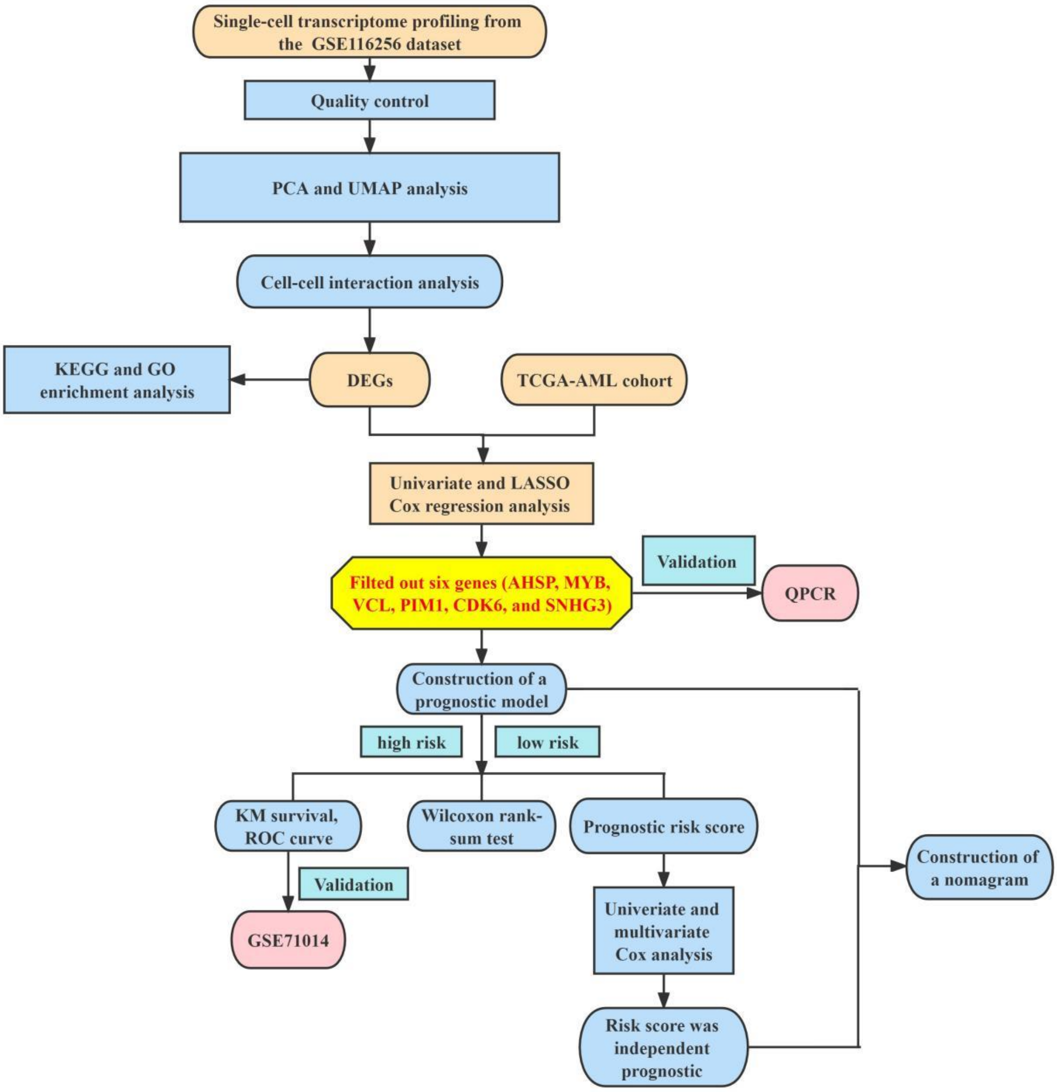

Supplementary Figure S1. The workflow of the research. Abbreviations are defined as follows, PCA: Principal Component Analysis; UMAP: Uniform Manifold Approximation and Projection; KEGG: Kyoto Encyclopedia of Genes and Genomes; GO: Gene Ontology; DEGs: Differential expressed genes; LASSO: least absolute shrinkage and the selection operator; KM: Kaplan-Meier; ROC: receiver operating characteristic.

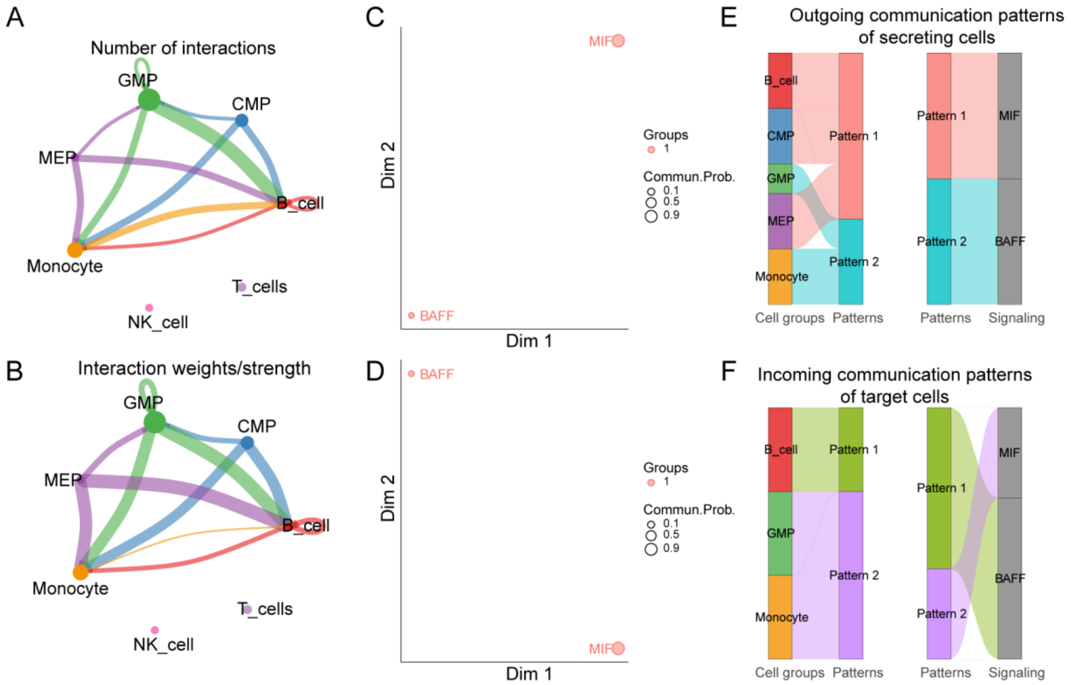

Supplementary Figure S2. The cell-cell communications analysis using CellChat. The aggregated cell-cell communication network for the numbers **(A)** and strength **(B)** of interactions. Projecting signaling pathways onto a two-dimensional manifold according to their functional **(C)** or structural similarity **(D)**. Each dot represents the communication network of one signaling pathway. Dot size is proportional to the overall communication probability. **(E)** The inferred outgoing communication patterns of secreting cells, indicating the correspondence between the inferred latent patterns, cell groups, and signaling pathways. **(F)** The inferred incoming communication patterns of target cells.

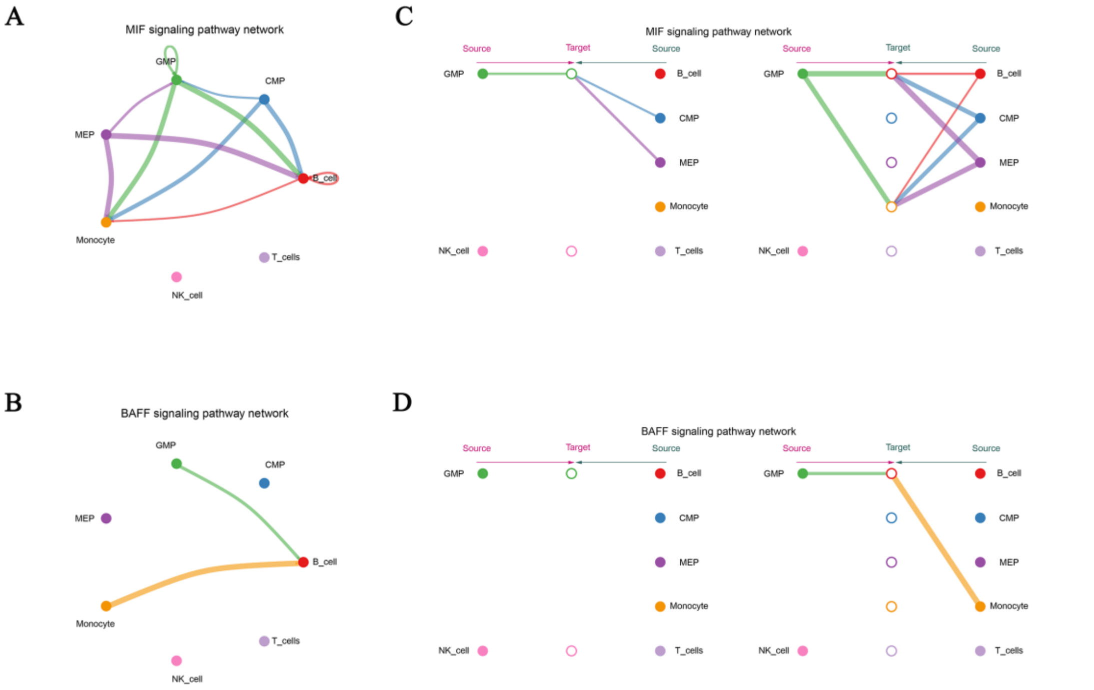

Supplementary Figure S3. Cell-cell interactions network in specific pathways. **(A)** The cell-cell interactions in MIF signaling pathway network. **(B)** The cell-cell interactions in BAFF signaling pathway network. **(C)** The MIF signaling pathway network in AML. **(D)** The BAFF signaling pathway network in AML.

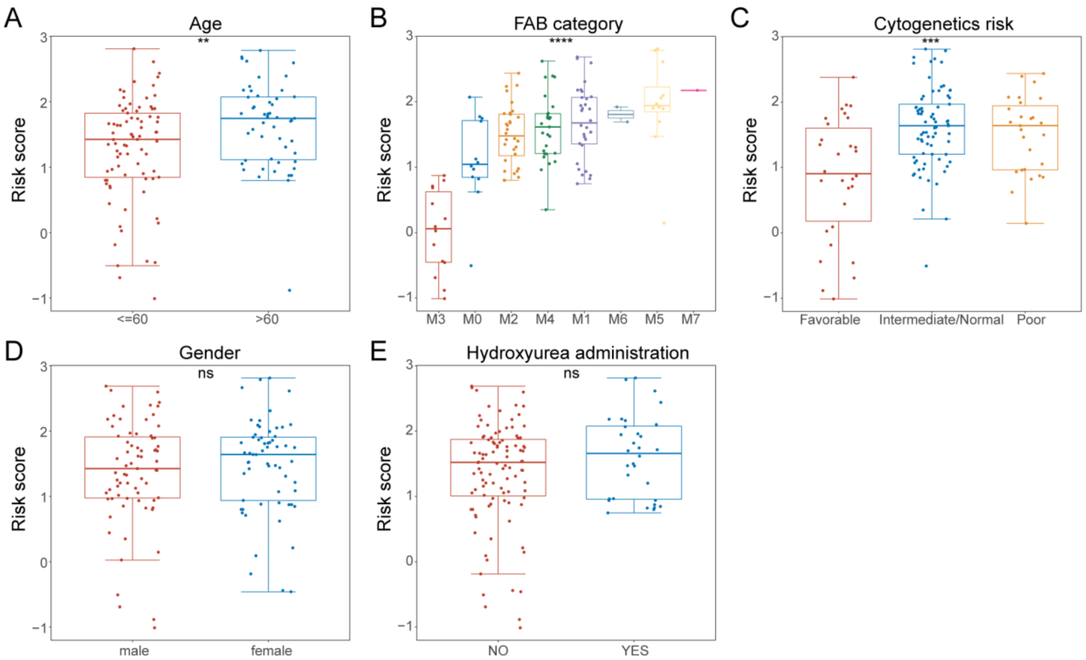

Supplementary Figure S4. Boxplots of risk score in different clinical sub-groups,

such as age **(A)**, FAB category **(B)**, Cytogenetics risk **(C)**, Gender **(D)**, Hydroxyurea administration**(E)**.

Supplementary Table S1. Detailed Summary of Patient Demographic Data.

Characteristics	Details
Number of patients	10 (paired samples: AML patients vs. healthy controls)
Age, median (range), years	31 (15–45)
Gender, n (%)	Male: 7 (70%); Female: 4 (30%)
AML subtype, n (%)	M1: 1(10%); M2: 2(20%); M4: 1 (10%); M5: 6 (60%) [based on FAB classification]
Clinical stage, n (%)	Newly diagnosed: 5(50%); Relapsed: 5(50%)
Treatment history, n (%)	Chemotherapy-naive: 5(50%); Previously treated: 5 (50%)
